# Supplementary material for: Causal Relationship between Aging and Anorexia Nervosa: A White-Matter-Microstructure-Mediated Mendelian Randomization Analysis
Source: Biomedicines. 2024 Aug 16;12(8):1874. doi: 10.3390/biomedicines12081874 (PMC11351342; doi:10.3390/biomedicines12081874)
Supplement: Supplementary file 1 [file biomedicines-12-01874-s001.zip › Supplementary Figure/Figure S8/Leaveoneout plot of MR analysis for TL to ubm-b-1554.pdf]

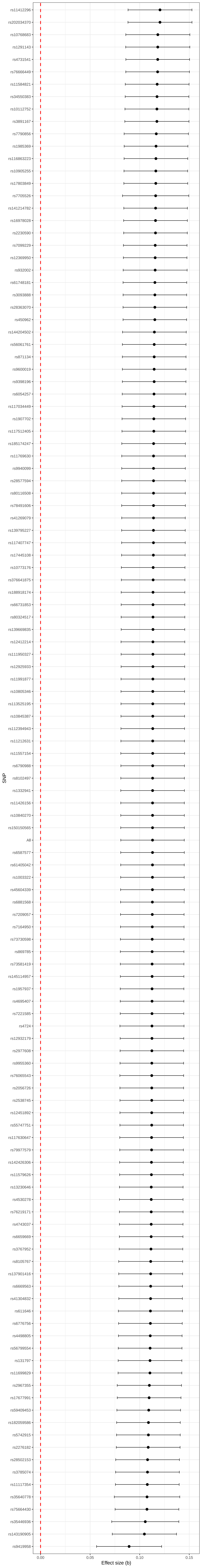
SNP
Effect size (b)
0.00
0.05
0.10
0.15
rs11412296
rs202034370
rs10768683
rs1291143
rs4731541
rs76666449
rs11584821
rs34550383
rs10112752
rs3891167
rs7790856
rs1985369
rs116863223
rs10905255
rs17803849
rs7705526
rs141214782
rs16978028
rs2230590
rs7099229
rs12369950
rs932002
rs61748181
rs3093888
rs28363070
rs450962
rs144204502
rs56061761
rs871134
rs9600019
rs9398196
rs6054257
rs117034449
rs1907702
rs117512405
rs185174247
rs11769630
rs9940099
rs28577594
rs80116508
rs78491606
rs41269079
rs139795227
rs117407747
rs17445108
rs10773176
rs376641875
rs188918174
rs66731853
rs80324517
rs139669835
rs12412214
rs111950327
rs12925933
rs11991877
rs10805346
rs113525195
rs10845387
rs112394943
rs11212631
rs11557154
rs6790988
rs8102497
rs1332941
rs11426156
rs10840270
rs150150565
All
rs6587577
rs61405042
rs1003322
rs45604339
rs6881568
rs7209057
rs7164950
rs73730598
rs869785
rs73581419
rs145114957
rs1957937
rs4695407
rs7221585
rs4724
rs12932179
rs2977608
rs9955360
rs76065543
rs2056726
rs2538745
rs12451892
rs55747751
rs117630647
rs79977579
rs142426306
rs11579626
rs13230646
rs4530278
rs76219171
rs4743037
rs6659669
rs3767952
rs8105767
rs137901416
rs6669563
rs41304832
rs611646
rs6776756
rs4498805
rs56799554
rs131797
rs11699829
rs2967355
rs17677991
rs59409453
rs182059586
rs5742915
rs2276182
rs28502153
rs3785074
rs11117354
rs35640778
rs75664430
rs35446936
rs143190905
rs9419958
